# Evaluation of Electrospun Poly‐4‐Hydroxybutyrate as Biofunctional and Degradable Scaffold for Pelvic Organ Prolapse in a Vaginal Sheep Model

**DOI:** 10.1002/mabi.202400412

**Published:** 2025-02-26

**Authors:** Krista L. C. van Rest, Stephen T. Jeffrey, Lisa Kaestner, Aksel Gudde, Anel Oosthuysen, Jan‐Paul W. R. Roovers, Zeliha Guler

**Affiliations:** ^1^ Amsterdam UMC Location University of Amsterdam Department of Obstetrics and Gynecology Amsterdam 1105 AZ The Netherlands; ^2^ Amsterdam Reproduction and Development Research Institute Amsterdam 1105 AZ The Netherlands; ^3^ Department of Obstetrics and Gynecology Groote Schuur Hospital University of Cape Town Cape Town 7925 South Africa; ^4^ Department of Urology Groote Schuur Hospital University of Cape Town Cape Town 7925 South Africa; ^5^ Cardiovascular Research Unit Division of Cardiothoracic Surgery University of Cape Town Cape Town 7925 South Africa

**Keywords:** absorbable scaffold, electrospinning, estradiol, poly‐4‐hydroxybutyrate (P4HB), vaginal sheep model

## Abstract

Pelvic organ prolapse (POP) affects many women, especially after menopause. POP occurs due to the descent of weakened supportive tissue. Current prolapse surgeries have high failure rates, due to disturbed wound healing caused by lower tissue regeneration and estrogen depletion. Absorbable poly‐4‐hydroxybutyrate (P4HB) knit implants exhibited improved cell and tissue response leading to less complications from prolapse surgery. This study aims to enhance wound healing and improve surgical outcomes by using an electrospun (ES) P4HB scaffold (ES P4HB) that emulates natural tissue structure. Further 17β‐estradiol (E2)‐a prominent wound healing factor‐is incorporated into the scaffold (ES P4HB‐E2). Parous Dohne Merino sheep underwent posterior vaginal wall implantation of either P4HB (*n* = 6) or 17β‐estradiol relasing P4HB‐E2 (*n* = 6) scaffolds, or underwent native tissue repair (NTR) (*n* = 4). Vaginal explants were compared for short‐term host response in terms of gross necropsy, histomorphology, biomechanics, tissue‐integration, and degradation of P4HB at 3‐months post‐implantation. Both scaffolds show promising results with enhanced mechanical properties and increased macrophage infiltration compared to NTR, but without differences between scaffolds. Thus, it seems electrospun P4HB scaffolds already improve tissue integration and healing. Further long‐term studies are needed before these scaffolds can be used in clinical practice.

## Graphical abstract

This study of a vaginal sheep model shows improved host response in vaginal tissue and enhanced biomechanical properties of electrospun absorbable poly‐4‐hydroxybutyrate (ES‐P4HB) scaffolds for pelvic organ prolapse surgery. It presents improvement of tissue integration and healing by the extracellular matrix (ECM)‐mimicking structure of ES‐P4HB with and without 17β‐estradiol‐releasing properties.

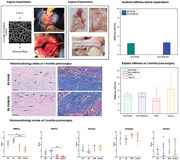

## 1 Introduction

Pelvic organ prolapse (POP), with risk factors being vaginal birth, age, and hypoestrogenism (e.g., postmenopausal status),^[^ ^1^, ^2^ ^]^ is a major health issue worldwide affecting ≈1 in 4 women. Earlier studies show a lifetime risk for surgery with the indication of POP up to 19%.^[^ ^3^, ^4^ ^]^ The primary surgical procedure to correct POP anatomically is native tissue repair (NTR). This surgical technique is based on a dissection of the vaginal wall, whereafter the prolapsed part is sutured onto a pelvic muscle or ligament. However, vaginal fibroblast function in POP patients is impaired and the tensile strength of the vaginal wall is lowered due to less and altered collagen and elastin fibers.^[^ ^5^ ^]^ Consequently, treatment of POP with NTR has high recurrence rates. As a result, permanent polypropylene (PP) implants have been used to induce a foreign body response (FBR). This results in the formation of new connective tissue that is load bearing against forces on the vaginal wall. Unfortunately, permanent vaginal implants have caused various long‐term complications, such as exposure, dyspareunia, bowel dysfunction, and de novo urinary incontinence. Therefore, an international ban for permanent mesh as primary care was issued.^[^ ^6^, ^7^ ^]^ As a result, there is an ongoing need for vaginal implants that temporarily adopt tissue strength, while enhancing tissue regeneration and wound healing, and result in lower recurrence rates than NTR. An alternative approach is the use of biological grafts, such as autologous, alloplastic, and homologous grafts (e.g., own fascial sheaths, porcine dermis, or cadaveric fascia lata).^[^ ^8^ ^]^ Although safe to use, biological grafts have not resulted in lower repeat surgery rates compared to NTR, presumably based on fast degradation rates.^[^ ^8^, ^9^, ^10^, ^11^ ^]^ As such, the Scientific Committee on Emerging and Newly Identified Health Risks (SCENIHR) advocated in their final opinion of December 2015: “further research for novel design and materials, in particular absorbable meshes, and improved technologies for manufacturing meshes, such as electrospinning.”^[^ ^12^ ^]^

Our group has been researching a fully absorbable material, poly‐4‐hydroxybutyrate (P4HB), with the hypothesis that its slow and gradual degradation, coupled with its mechanical strength, will elicit a mild host response with less adverse events compared to permanent mesh. In fact, we have reported improved vaginal fibroblast activity in vitro on the cells cultured on knit P4HB, excellent tissue integration with dense connective tissue, a moderate host response, and improved biomechanics following vaginal implantation of P4HB in sheep at 6 months follow‐up^[^ ^13^ ^]^ and mechanically self‐sufficient tissue with no adverse events after complete degradation of the knit P4HB in sheep at 24‐months.^[^ ^14^ ^]^ Based on these encouraging results on the knit P4HB implant, we created an electrospun (ES P4HB) scaffold to further improve its performance as electrospun scaffolds serves high surface‐to‐volume ration and high porosity which favor scaffold‐cell interactions and host response.^[^ ^15^ ^]^ We have demonstrated that ES P4HB resulted in higher cell proliferation, collagen‐ and elastin deposition in vitro as compared to knit design.^[^ ^15^ ^]^ Electrospinning is a technique that leverages electric potential to generate ultrathin polymeric fibers, ranging from the micro to nanoscale. It allows not only the production of porous scaffolds that can mimic extracellular matrix (ECM), but also enables the biofunctionalization of the scaffold by embedding bioactive compounds which results in scaffolds that serve as a controlled delivery system. For POP treatment different approaches of compound delivery via ES scaffolds were studied, such as degradable mesh releasing basis fibroblast growth factor (bFGF), connective tissue growth factor (CTGF), mesenchymal stem cells (MSC), and cyclic arginine‐glycine‐aspartic acid (cRGB).^[^ ^16^, ^17^, ^18^, ^19^ ^]^ However, these studies only evaluated the scaffolds within in vitro conditions or small animal studies with abdominal models which have low translatability to clinical practice.

In our study, we therefore chose a large animal vaginal model, and an estrogen‐enhancement of the scaffold. Vaginal admission of estrogen has been shown to decrease pH and promote vaginal cell maturity,^[^ ^20^ ^]^ restore altered vaginal angioarchitecture^[^ ^21^ ^]^ and increase production of new extracellular matrix.^[^ ^22^ ^]^ Furthermore, a recent meta‐analysis concluded that administration of estrogen before and/or after vaginal surgery specifically, could enhance vaginal wound healing by a reduced inflammatory response and increased vascularization, tissue granulation, wound closure, and collagen formation.^[^ ^23^ ^]^ We hypothesize that controlled release of estrogen from ES P4HB at the surgical site can improve surgical outcomes by improving tissue regeneration and wound healing. Thus, we modified ES P4HB with 17β‐estradiol (E2) and created a hormone‐releasing scaffold (ES P4HB‐E2). The addition of E2 to the scaffold did not alter the microstructure of the implant. Also, a burst release of E2 for 7 days with a linear sustained release thereafter for the full 28 days study period was achieved.^[^ ^15^ ^]^

As a next step, in this study we aim to i) compare the short‐term host response and biomechanics of the ES P4HB and ES P4HB‐E2 following vaginal implantation in vivo in sheep and ii) assess the added value of estrogen on vaginal wound healing in the sheep vaginal model.

## 2 Experimental Section

### 2.1 Setting

This study was conducted at the Mariendahl Experimental Farm, which is a university facility for the Department of Animal Science of the University of Cape Town (UCT). Guidelines for the care and use of laboratory animals of the National Health and Medical Research Council of South Africa were followed here, including daily monitoring and weekly weighing of sheep. The Animal Ethics Committee of the UCT approved the experimental protocols (AEC #022_010) for the maintenance and treatment of the sheep. Only sheep were used in the study; patients were not involved. Animal recruitment, surgical procedures, and housing were executed at Mariendahl. All animals were housed outdoors for at least 7 days, until 3 days prior to surgery. Then, animals were held in pens within pairs or groups, never alone, with free access to food and water and no movement restrictions. After surgery, animals stayed at the farm under the same conditions, with the benefit of no unnecessary stress inducement through transportation.

### 2.2 Materials

In this study, electrospun poly‐4‐hydroxybutyrate (ES P4HB) and 17β‐estradiol (2%) releasing P4HB (ES P4HB‐E2) were used, and scaffold selection was made based on the in vitro study.^[^ ^15^ ^]^ Textural properties such as fiber diameter, pore size, thickness and porosity, the mechanical properties and the water contact angle of the scaffolds were determined before the implantation. The average fiber diameters and pore sizes of the scaffolds were calculated from SEM images using ImageJ (v1.52q, NIH Image, USA). The porosity of the scaffolds was measured using the differential mass of the scaffold in air versus the scaffold mass in heptane. See the Supporting Information S1 for the details of porosity calculation.

### 2.3 Study Design and Sample Size

Sixteen parous female Dohne Merino sheep of ≈5–6 years old, with a mean weight of 66.58 ± 7.3 kg, were studied for a follow‐up period of 3 months. All sheep were randomly selected, assigned a number, and divided into pens. Thereafter, sheep were randomly divided into three groups; ES P4HB (*n* = 6), ES P4HB‐E2 (*n* = 6), and NTR (*n* = 4) (see the Supporting InformationS2 for the sample size calculation). To reduce the animal number, middle vaginal tissue (vaginal control) was collected from the same sheep as controls.

### 2.4 Surgical Procedures

**Figure 1:**
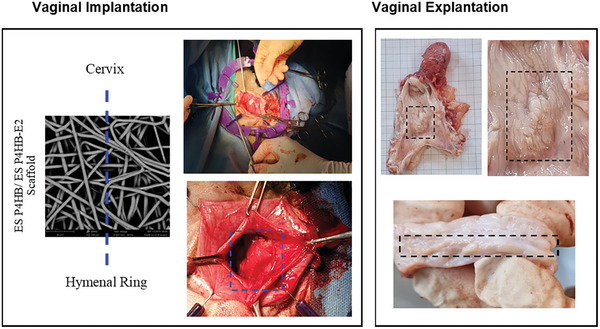
Vaginal implantation of ES P4HB or ES P4HB‐E2 scaffolds in the posterior compartment and explantation images of the vaginal tissue showing the healed incision and cross‐sectional view of scaffold‐tissue integration.

All sheep had vaginal wall reconstruction of the posterior compartment with ES P4HB and ES P4HB‐E2 scaffolds (35 mm x 35 mm) or NTR surgery (**Figure** **1**). All animals were sedated before general anesthesia with intubation was admitted. The surgeon was blinded to the treatment group and thus performed NTR unless the present researcher provided an implant. The rectovaginal septum was dissected by aqua‐dissection, and (scaffold) space was bluntly and sharply created between the vaginal epithelium and rectal serosa above the hymeneal ring. If indicated, dry scaffolds were fixed with 3‐0 prolene sutures, and for NTR fascial structure was plicated with three interrupted 3/0 polyglactin 910 (Vicryl, Ethicon, Raritan, USA) sutures. The vaginal walls of all groups were closed with running 3/0 polyglactin 910 (Vicryl) suture. Thereafter, a vaginal tampon was inserted for 24 h. For a more detailed version of the procedure please see the Supporting Information S3.

### 2.5 Explantation and Anatomical Evaluation

Animals were euthanized at 3 months after surgery with intravenous 50 mL of Sodium Pentobarbitone, after receiving premedication with midazolam 0.3 mg kg^−1^ and ketamine 3 mg kg^−1^ intravenously and Isoflurane inhalation (1%–2%) for short general anesthesia. Thereafter, vagina was excised en bloc and gross anatomical examination was performed before the dissection of the explants (vaginal tissue and scaffold complex) and NTR and vaginal controls. Gross anatomical examination was performed for the following parameters: (I) fluid collection, (II) exposure of the implant, (III) synechiae, and (IV) signs of infection. Full‐thickness tissues of 3 × 3 cm were collected, and each tissue sample was divided into four pieces for uniaxial tensile testing, in vivo degradation, histology, and scanning electron microscopy.

### 2.6 Outcome Measures

#### 2.6.1 Biomechanics of the Explants

The biomechanical properties of the ES P4HB and ES P4HB‐E2 vaginal explants together with NTR and middle vaginal tissue as control were determined with uniaxial tensiometry by using Instron 5544 (Norwood, MA) with a 200N load cell. In addition, mechanical properties of the scaffolds before the implantation were measured as well. Scaffolds or explants were cut longitudinally (10 × 30 mm), clamped tension free, and the zero elongation was defined as the clamp‐to‐clamp distance at preload (0.1 N). The load was applied with an elongation rate of 10 mm min^−1^ until failure. The strain was calculated by dividing the elongation by the clamp‐to‐clamp distance and stress by dividing the force by the cross‐sectional area of the sample before testing. The stiffness (N mm^−1^) of the specimens was determined with the slope of the stress–strain curve in the comfort zone by using OriginPro2018 software (OriginLab Corporation, Northampton, MA).^[^ ^14^, ^24^ ^]^

#### 2.6.2 In Vivo Degradation

The in vivo degradation of the P4HB scaffold was determined by defining the amount and structure of the remaining scaffold in the tissue. The molecular weight (Mw) change was assessed through gel permeation chromatography (GPC) analysis. Meanwhile, the morphological changes in the scaffold were examined via scanning electron microscopy (SEM) (JEOL JSM6700F). The vaginal tissue was removed from the explants using tissue digestion with collagenase to identify the amount of remaining implant in the tissue over time.^[^ ^14^ ^]^ For the details of tissue removal, and GPC and SEM analyses, see the Supporting Information S4.

#### 2.6.3 Histomorphology

A detailed description of methods of staining and scoring are provided in the Supporting Information S5. Specimens were scored (0–3) for presence of foreign body giant cells (FBGC), polymorphonuclear cells (PMN), vessels, collagen through Hematoxylin and Eosin (H&E). Masson's trichrome and Verhoeff‐Van Gieson's staining were used for presence of collagen and elastin, respectively (score: 0–4). With immunohistochemistry (IHC) staining, the presence of smooth muscle (αSMA), neovascularization (CD34), macrophages type I (HLA‐DR), and macrophages type II (CD‐163) were analyzed. Score ranges for IHC lay between 0 and 4. (For details see the Supporting Information S5).

#### 2.6.4 Scaffold‐Tissue Integration

Integration of the ES P4HB and ES P4HB‐E2 scaffolds was evaluated from the SEM images of the explants following tissue dehydration in gradually increasing concentrations of alcohol solutions (0%–100%) and hexamethyldisilazane (HDMS) (For details see the Supporting Information S6).

### 2.7 Statistical Analysis

Statistical analysis was performed with GraphPad Prism 9.5.1 (GraphPad Software, San Diego, CA). Data normality was tested by Shapiro–Wilk test. A two‐way analysis of variance (ANOVA) was used for normally distributed data and multiple comparisons between individual groups were made using Tukey's test. The Mann–Whitney U test or Kruskal–Wallis test followed by Dunn's post hoc test was used for not normally distributed data. Data are reported as means ± standard deviations or standard errors of the mean, or medians with interquartile ranges. The significance level was defined as *p* < 0.05.

### 2.8 Ethical Statement

This study was conducted with approval of The Animal Ethics Committee of the University of Cape Town (AEC #022_010), and animals were maintained and treated according to experimental protocols (Code of Ethics and Procedures for the Use of Animals in Teaching and Research, University of Cape Town /AEC SOP).

## 3 Results

### 3.1 Before Implantation

#### 3.1.1 Textural Properties and Water Contact Angle of the Scaffolds

**Figure 2:**
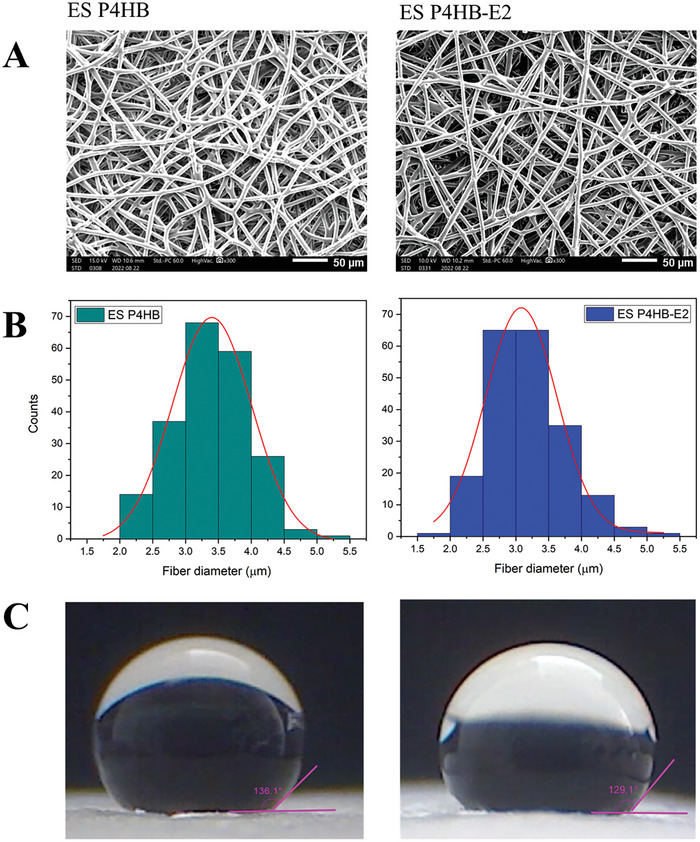
Scanning electron microscopy (SEM) images A), fiber size distribution B), and water contact angle C) of ES P4HB and ES P4HB‐E2 scaffolds. The scale bars indicate 50 µm.

The textural properties of both ES P4HB and ES P4HB‐E2 scaffolds were comparable (**Table** **1** and **Figure** **2**A,B). The addition of estradiol to the ES P4HB scaffold structure did not interfere with their textural properties. Water contact angle values (Figure 1C) of the ES P4HB and ES P4HB‐E2 were measured as 136.1 ± 2.7 and 129.1 ± 1.5 (*p* < 0.05), respectively, which shows the hydrophobic nature of the scaffolds.^[^ ^25^ ^]^

**Table 1:** Textural properties of the ES P4HB and ES P4HB‐E2.

	ES P4HB	ES P4HB‐E2
Fiber diameter [µm]	3.4 ± 0.6	3.2 ± 0.6
Pore size [µm^2^]	13.5 ± 1.3	15.3 ± 1.2
Porosity [%]	76.1 ± 0.7	75.1 ± 0.6
Thickness [µm]	497.7 ± 47.3	574.7 ± 41.2

#### 3.1.2 Mechanical Properties of the Scaffolds

**Figure 3:**
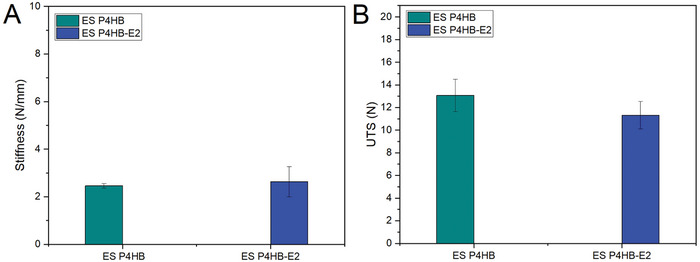
Stiffness A) and ultimate tensile strength (UTS) B) of ES P4HB and ES P4HB‐E2 scaffolds before implantation. Error bars represent means ± standard deviations (SD). Two‐way ANOVA and multiple comparisons between individual groups using Tukey's test were used to test for differences between groups and time points. Values differing significantly from the control are indicated by asterisks: *p < 0.05.

ES P4HB and ES P4HB‐E2 scaffolds exhibited comparable mechanical properties before the implantation (**Table** **2** and **Figure** **3**). The stiffness of the ES P4HB and ES P4HB‐E2 was determined as 2.5 ± 0.1 and 2.6 ± 0.6 N mm^−1^, respectively (Figure 3A).

**Table 2:** Mechanical properties of the ES P4HB and ES P4HB‐E2 scaffolds before the implantation, and mechanical properties of the ES P4HB and ES P4HB‐E2 explants at 3‐months postimplantation compared with NTR and vaginal controls.

	Before implantation		3‐months postimplantation			
	ES P4HB	ES P4HB‐E2	ES P4HB	ES P4HB‐E2	NTR	Vaginal control
Stiffness [N mm^−1^]	2.5 ± 0.1	2.6 ± 0.6	5.1 ± 3.0	5.3 ± 3.6	3.7 ± 2.2	6.5 ± 3.4
UTS [N]	13.1 ± 1.4	11.3 ± 1.2	37.2 ± 23.0	42.8 ± 14.0	28.5 ± 4.4	36.7 ± 16.5
Ultimate elongation [%]	278.4 ± 12.0	279.0 ± 15.0	231.0 ± 96.3	176.0 ± 58.9	211.6 ± 34.6	183.6 ± 22.5

### 3.2 After Implantation

#### 3.2.1 Gross Necropsy

**Figure 4:**
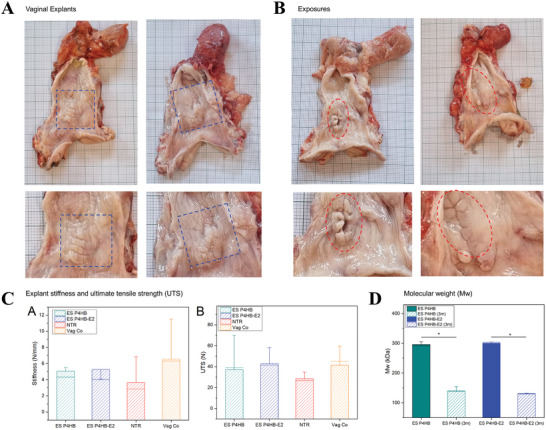
Explant properties; gross necropsy showing vaginal explants A) and exposures B), biomechanics C), and in vivo degradation D). (A) shows representative images of vaginal explants and (B) shows exposures through the incision site. (C) shows stiffness (A) and ultimate tensile strength (UTS) (B) of ES P4HB and ES P4HB‐E2 explants at 3‐months postimplantation compared with NTR and vaginal control tissue (tissue harvested from posterior middle vagina). (C) shows molecular weight (Mw) of ES P4HB and ES P4HB‐E2 scaffolds before and 3‐months (3 m) after implantation. Error bars represent means ± standard deviations (SD). Two‐way ANOVA and multiple comparisons between individual groups using Tukey's test were used to test for differences between groups and time points. Values differing significantly from the control are indicated by asterisks: *p < 0.05.

ES P4HB and ES P4HB‐E2 were incorporated well in the vaginal tissue with no sign of encapsulation (**Figure** **4**A). In addition, no fluid collections, synechiae, or signs of infection were observed in all animals. However, exposure at the incision site was seen in the ES P4HB (*n* = 1/6, size of the exposure: 1 mm × 2 mm) and the ES P4HB‐E2 group (*n* = 2/6, size of the exposure: 2 mm × 20 mm and 5 mm × 20 mm). When treatment groups are compared, no statistical differences were found for exposure (*p* > 0.99).

#### 3.2.2 Biomechanics of the Explants

The biomechanical properties of the vaginal ES P4HB and ES P4HB‐E2 explants, NTR and vaginal control tissue were compared at 3‐months postimplantation (Table 2 and Figure 4B). Although not statistically significant (NS), the stiffness of both ES P4HB (5.1 ± 3.0 N mm^−1^) and ES P4HB‐E2 (5.3 ± 3.6 N mm^−1^) explants was higher than of NTR tissue (3.7 ± 2.2 N mm^−1^) but lower than of vaginal controls (6.5 ± 3.4 N mm^−1^). In addition, ultimate tensile strength (UTS) values of both explants were higher than of NTR and comparable to vaginal control tissue (NS). The explant stiffness and UTS of ES P4HB and ES P4HB‐E2 were comparable at 3‐months.

#### 3.2.3 In Vivo Degradation

The degradation of the P4HB scaffolds was determined by the change in its molecular weight (Mw) over time (Figure 4C). The average Mw of the ES P4HB and ES P4HB‐E2 scaffolds before implantation was 295.8 ± 7.8 and 300.4 ± 5.8 kDa, respectively. The addition of E2 did not interfere with the Mw of the scaffold. After 3 months postimplantation, Mw of both ES P4HB and ES P4HB‐E2 decreased statistically significantly to 138.5 ± 17.0 kDa (*p* = 0.00) and 129.6 ± 3.4 kDa (*p* = 0.00), respectively. This indicates that more than half of the scaffolds (ES P4HB 46.8% and ES P4HB‐E2 43.1%) were degraded after 3‐months. Although ES P4HB‐E2 scaffolds degraded slightly faster than ES P4HB, the difference was not statistically significant.

#### 3.2.4 Tissue Integration and Histomorphology

**Figure 5:**
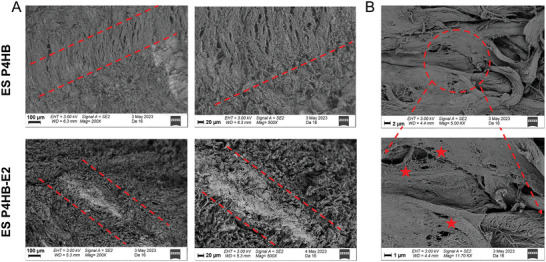
Scanning electron microscopy images of ES P4HB and ES P4HB‐E2 explants in low (the scale bar is 100 µm) (left) and high magnification (the scale bar is 20 µm) (right) showing the scaffold‐tissue integration at 3‐months postimplantation A) and nanofibrous structure of the scaffold that mimics the tissue ECM structure in high magnification (the scale bars are 2 µm and 1 µm) B). Arrows indicate the placement of the scaffold within the vaginal tissue and stars indicate the fibrous structure of the scaffolds.

SEM images show that both scaffolds were well integrated with the vaginal tissue (**Figure** **5**A) and the fibrous, porous structures of the scaffolds are mimicking the structure of the ECM components of the tissue (Figure 5B). This enhances the scaffold‐tissue interaction.

**Figure 6:**
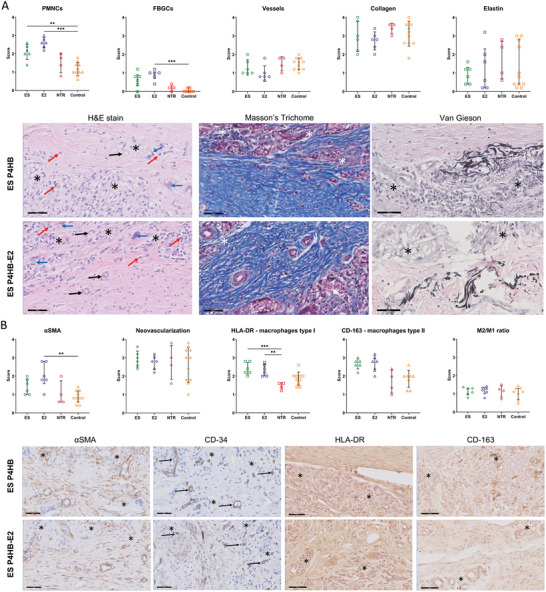
Mean and median scores for presence of A) PMNCs, FBGCs, vessels, collagen, and elastin, and B) smooth muscle cells (αSMA), neovascularization (CD‐34), macrophages types I (M1) and type II (M2) and M1/M2 ratio. Error bars represent means ± standard deviations (SD) or median ± interquartile range. Two‐way ANOVA and Tukey's multiple comparisons test between individual groups were used to test for differences between groups. ** indicates *p* < 0.01, *** indicates *p* < 0.001. All statistical results with *p* < 0.05 are written out in text in the manuscript. Images show representative histomorphology slides, with indication as follows; * = scaffold location, blue arrow = FBGC, black arrow = vessel, red arrow = PNMC.

Host responses to all groups (ES P4HB, ES P4HB‐E2, NTR, and vaginal control) at 3 months postimplantation were compared (**Figure** **6**). Both ES P4HB and ES P4HB‐E2 have higher immune cell infiltration evidenced by higher scores for PMNCs compared to vaginal control, with respectively mean differences of 1.0 (95%CI 0.3–1.7, *p* < 0.01) and 1.5 (95%CI 0.8–2.2, *p* < 0.01) (Figure 6A). Furthermore, ES P4HB‐E2 results in more PMNC infiltration when compared to NTR, with a mean difference of 1.0 (95%CI 0.2–1.9, *p* < 0.01). There was mild presence of FBGCs around the ES P4HB and ES P4HB‐E2 scaffolds with significantly higher infiltration as compared to vaginal control (0.7 IQR 0.3–0.9 for ES P4HB, 1.0 IQR 0.7–1.1 for ES P4HB‐E2, and 0 IQR 0–0.2 for controls, resp. *p* < 0.03/<0.01). There was large presence of collagen (score 3/4) and mild elastin formation (score 1/4) around the scaffolds, however there was no statistical difference for collagen and elastin formation between the groups at 3‐months postimplantation (Figure 6A). Myofibroblast differentiation values were significantly higher around the ES P4HB‐E2 scaffold (score 1.4 IQR 1.0–1.9) than vaginal control (score 0.8 IQR 0.6–1.2, *p* < 0.01) (Figure 6B). High neovascularization was found in all groups. There were statistically significantly more M1 present around the ES P4HB (2.4 ± 0.3SD) and ES P4HB‐E2 (2.3 ± 0.3SD) scaffolds than vaginal control (1.9 ±0.4SD; ES P4HB vs control *p* < 0.02 and ES P4HB‐E2 vs control *p* < 0.05) and NTR (1.5 ±0.2SD, ES P4HB vs NTR *p* < 0.01 and ES P4HB‐E2 vss NTR *p* < 0.01). The presence of M2 (ES P4HB: 2.7 ± 0.3SD and ES P4HB‐E2: 2.7 ±0.4SD) was higher than M1 and there were statistically significantly more M2 around the scaffolds than vaginal control (1.9 ± 0.5SD; ES P4HB vs control *p* < 0.01; ES P4HB‐E2 vs control *p* < 0.01) and NTR (1.7 ±0.6SD; ES P4HB *p* < 0.02 and ES P4HB‐E2 *p* < 0.01). There were no statistical differences between M2/M1 ratios of the experimental groups and large presence of neovascularization was seen in all groups (NS, Figure 6B).

## 4 Discussion

In this study, we compared the short‐term host response and biomechanics of electrospun P4HB scaffolds (ES P4HB) and 17β‐estradiol (E2) modified scaffold (ES P4HB‐E2) in a vaginal sheep model and found no statistical differences between these scaffolds. Also, we compared the scaffolds to NTR and vaginal control tissue. In this comparison, both scaffolds with and without E2 improved the tissue biomechanics (NS) demonstrated by higher stiffness and ultimate strength compared to vaginal controls, despite the significant degradation (≈50%) of the scaffolds. ES P4HB and ES P4HB‐E2 both exhibited good integration with the vaginal tissue and resulted in moderate host response with high collagen deposition in all groups and higher immune response as compared to NTR and vaginal control. The addition of E2 did not lead to statistically superior results.

The textural properties of the scaffolds were comparable and the addition of E2 into scaffold structure did not interfere with the textural and mechanical properties of the scaffold. The stiffness of the scaffolds is strongly related to both density and porosity of the implant,^[^ ^24^, ^26^ ^]^ which might explain the comparable mechanical properties of both scaffolds. The in vivo degradation of both scaffolds was also similar to each other and was not affected by the E2 addition.

### 4.1 Improved Biomechanics Despite Degradation of the Scaffolds

3‐months postimplantation, both explants degraded gradually over time and half of the scaffolds were absorbed. These findings are comparable with our earlier in vitro and in vivo results.^[^ ^13^, ^15^ ^]^ P4HB has an advantage over other degradable polymers like polyglycolide, as it releases its degradation products gradually and therefore loses its mechanical properties slowly.^[^ ^27^ ^]^ Through the slow but steady degradation process of the P4HB, the surrounding tissue was able to remodel and achieve enough tensile strength to take over the mechanical load bearing of the scaffold. Fast degradation may result in recurrence of POP, therefore slow degradation of electrospun P4HB may be advantageous in the long term.^[^ ^14^, ^28^ ^]^ For other electrospun scaffolds based on ureidopyrimidinone polycarbonate (UPy‐PC) and polycaprolactone (PCL) the degradation processes in sheep were faster and concerns were raised to whether these scaffolds suffice in supporting the newly developed tissue.^[^ ^16^, ^29^, ^30^ ^]^ Even though electrospun P4HB scaffolds with or without E2 degrade gradually and allow for new tissue regeneration and growth, they degrade faster than knit P4HB. We showed this in a comparable sheep study; 30% of knit P4HB was degraded at 3‐months postimplantation (the average Mw of the knit P4HB reported as: 279 ± 3, 201 ± 5, and 104 ± 7 kDa SD at 0, 60, and 180 days postimplantation, respectively), whereas almost 50% of ES scaffolds was degraded in this study.^[^ ^13^ ^]^ Still, compared to other degradable polymers, it is safe to say P4HB is a slowly degradable polymer which allows gradual load transfer from the scaffold to the vaginal tissue.

The load transfer from scaffold to vaginal tissue is evidenced by higher (almost double) explant stiffness as compared to scaffold stiffness. The explant stiffness results from the combination of the stiffness of the implant, the underlying grafted vagina, and newly ingrown tissue.^[^ ^14^, ^31^ ^]^ Considering the scaffold stiffness values were around 2 N mm^−1^, while the ones for explants were around 5 N mm^−1^ and only half of the scaffolds are remaining in the vaginal explants, the contribution of the scaffold to the explant stiffness is limited. In addition, it should be noted that the UTS values of the explants are dramatically higher than the ones of scaffolds before the implantation. As a result, we can infer that the mechanical support of the explants comes from remodeling and newly formed ingrown tissue.

### 4.2 Gross Necropsy

No infections, fluid collections, or synechiae were observed after the implantations of both scaffolds, thus showing good integration in the vaginal tissue. However, exposure at the incision site was observed in both ES P4HB (*n* = 1/6) and ES P4HB‐E2 (*n* = 2/6) groups. This might be related to the thickness of the scaffolds as both electrospun scaffolds exhibit comparable stiffness values to each other and to a previously evaluated knit P4HB scaffold.^[^ ^13^ ^]^ This scaffold however did not result in any exposures.^[^ ^13^, ^24^ ^]^

The high thickness and porosity of the scaffolds might have resulted in mesh burden leading to exposures—a finding that aligns with observations made in women.^[^ ^32^ ^]^ Animal studies have shown higher exposure rates compared to those in women, which could be attributed to the varying vaginal environments, the skill level of the surgeon, and potentially the use of more experimental implants in animal research. Also, larger animals tend to have higher exposure rates than smaller ones. This could be due to the use of larger implants in bigger animals.^[^ ^33^ ^]^ In addition, compared to studies with enhancement of the scaffolds with CTGF, MSC, and/or bFGF, it is noted that the addition of estradiol has not led to herniation, tumor, or abscess formation.^[^ ^16^, ^17^, ^19^ ^]^ This could be due to the location of the implant (abdominal vs vaginal) and the size of the implant.

Still, degradable implants are less stiff and show less deformation after implantation,^[^ ^29^, ^34^, ^35^ ^]^ which leads to less exposure and pain.^[^ ^36^ ^]^ In addition, it should also be noted that P4HB is a fully absorbable material and has potential to eliminate adverse events in the long term. Our earlier performed 2‐year follow up study on knit P4HB implants confirms no adverse events including exposures after complete degradation of the P4HB.^[^ ^14^ ^]^

### 4.3 Scaffolds Induced Host Response and Wound Healing

ES P4HB and ES P4HB‐E2 scaffolds resulted in a moderate host response with elevated levels of early phase FBR. For example, the infiltration of PMNCs and FBGCs at the interface of the ES P4HB and ES P4HB‐E2 scaffolds were higher compared to NTR and vaginal control tissue. It is known that electrospun implants/scaffolds tend to trigger FBR more than knit implants due to their nonwoven and porous structure.^[^ ^29^ ^]^ The electrospun scaffolds' high surface area, volume ratio, and porosity mimic the natural ECM structure, providing an extensive and accessible fiber surface for cell and tissue interactions. Scaffold pore size plays a crucial role in determining whether cells perceive the scaffold as 2D or 3D. Electrospun P4HB scaffolds exhibit pore sizes exceeding 8 µm, meeting the requirement for cellular ingrowth. Additionally, the porosity of the scaffolds exceeds 70%, enabling cell migration through the scaffold while providing adequate space for nutrient delivery and waste removal.^[^ ^15^, ^37^, ^38^, ^39^ ^]^ The nanoscale organization of the electrospun scaffolds, which mimics the ECM, can facilitate cell behavior and tissue structure development. The larger surface area allows for protein absorption and offers binding sites for cell membrane receptors. Consequently, fibroblasts integrate more effectively, and a more healing FBR is achieved.^[^ ^40^ ^]^ The upregulation of vaginal fibroblast function and initiation of ECM turnover is accompanied by collagen and elastin deposition.

In this study, both scaffolds exhibited high collagen levels, although not statistically significantly different than controls, and the presence of elastin demonstrated that fibroblast function was not impaired. The higher collagen and elastin deposition might indicate mechanically stronger and more elastic tissue. Earlier animal studies of knit P4HB showed less fast, but abundant elastin formation.^[^ ^14^, ^41^ ^]^ The relatively early observation of the elastin in our vaginal explants after implantation of the ES P4HB and ES P4HB‐E2 scaffolds might be related with the ECM mimicking nature of the electrospun scaffolds.

In proper tissue healing M1‐like proinflammatory macrophages dominate the early stages of the process and M2‐like macrophages contribute to tissue healing and regeneration during the resolution phase. The inflammatory response decreases over time.^[^ ^13^, ^14^ ^]^ In this study, M1 response of both scaffolds was higher than NTR. Considering that the M2 response to the ES P4HB and ES P4HB‐E2 scaffolds was higher than M1 response at 3‐months and the M2/M1 ratio of the scaffolds was balanced (M2/M1), we conclude that the tissue regeneration and healing was promoted within 3 months after implantation. While initial research categorized macrophages into distinct M1 (proinflammatory) and M2 (anti‐inflammatory) phenotypes, current understanding recognizes this might be an oversimplification. Because macrophages demonstrate significant plasticity in response to environmental signals resulting in a spectrum of immunophenotypes with overlapping functions. Although alternative classification systems have been proposed for animal models, the M1/M2 paradigm remains valuable for understanding immunopathological processes and developing therapeutic strategies, despite its’ limitations.^[^ ^42^, ^43^ ^]^

Furthermore, both scaffolds exhibited a mild‐moderate myofibroblast activity at short term. Myofibroblast differentiation was significantly higher in ES P4HB‐E2 scaffolds as compared to vaginal control tissue, while there was no significant difference between ES scaffolds. Myofibroblasts activity in early phase of healing might be related with the proper wound healing and ECM up‐regulation.^[^ ^5^ ^]^ Therefore, higher α‐SMA activity of the ES P4HB‐E2 might be contributing to the high collagen levels and improved mechanical strength of the vaginal tissue. However, myofibroblast activity of the ES scaffolds should be followed in long term, since high presence of myofibroblasts after the remodeling phase might be associated with adverse events, such as fibrosis or exposure.^[^ ^5^, ^13^, ^44^, ^45^ ^]^

### 4.4 Additional Effect of Estrogen

In this study, we did not observe statistically significant differences between ES P4HB and ES P4HB‐E2, although biofunctionalization of P4HB scaffold with E2 led to slightly more pronounced FBR in terms of higher PMNCs, FBGCs, M1‐like macrophages, and α‐SMA as compared to vaginal control or NTR tissue. The addition of E2 to the scaffold did not interfere with textural properties, in contrast to other studies where addition of estrone to ES poly (lactic‐*co*‐glycolic acid) nanofibers led to lower diameter of fibers. However, simultaneously, it did increase hydrophilicity that could promote cell adhesion and proliferation.^[^ ^46^ ^]^

E2 was incorporated into ES P4HB structure as it is considered beneficial to multiple aspects of wound healing, such as closure, neovascularization, and collagen synthesis.^[^ ^23^ ^]^ In this study, we used 2% E2 containing ES P4HB scaffolds based on our earlier in vitro study that showed ES P4HB‐E2 to steady release E2 over time and to promote early elastin deposition, as well as collagen‐I and III expression and matrix metalloproteinase‐type 2, therefore supporting ECM deposition and remodeling.^[^ ^15^ ^]^ However, we found that the used E2 concentration may not be optimal to result in statistically significant differences compared to the P4HB scaffold without E2 in in vivo conditions. For example, both scaffolds exhibited high vascularization (NS). This is not in line with an earlier study of ES estradiol‐releasing PU scaffolds in an ex ovo chick chorioallantoic membrane model that induced a higher number of blood vessels radiating to the scaffold compared to control ES PU scaffolds.^[^ ^47^ ^]^ More contradicting results are reported in literature. In favor of E2, more ECM production in stem cells on the E2‐releasing electrospun PU scaffolds was reported.^[^ ^47^ ^]^ Also, ADMSCs cultured on estradiol‐releasing PLA meshes produced more ECM involving collagen I, collagen III, and elastin, and new blood vessel formation in the chorioallantoic membrane was doubled.^[^ ^22^ ^]^ In contrast, it was reported that E2 suppresses proliferation of the POP fibroblasts.^[^ ^48^ ^]^ The difference in the reported effect of E2 might be related with the high heterogeneity of the studies, such as route of administration, used concentrations, differences in the experimental settings and follow‐up. Therefore, future study designs should account for these between‐study differences and optimal E2 concentrations should be optimized. While we did observe some beneficial effects of the E2, the optimum concentration of estrogen should be studied in vitro to achieve more pronounced and consistent effects on inflammatory as well as proliferative and remodeling components of wound healing.^[^ ^23^ ^]^ Despite these findings, both the ES P4HB and ES P4HB‐E2 significantly influenced the host response and the FBR, as clearly demonstrated by the augmented mechanical properties, increased immune cell infiltration, elevated collagen levels, and an optimally balanced M2/M1 ratio. From these observations, it could be inferred that the integration of E2 may not be an absolute necessity. This is because the ES P4HB scaffolds, in their existing state, already enhance tissue integration and healing processes. This enhancement can be attributed to their structure that closely mimics the ECM. However, it is important to note that before these electrospun P4HB (E2) scaffolds can be adopted for clinical practice, long‐term preclinical follow‐up studies, and clinical safety studies are required. These studies should aim to provide substantial evidence supporting the beneficial impacts of E2 and electrospun P4HB scaffolds on tissue integration and healing.

### 4.5 Limitations and Strengths

Some limitations of the study have to be addressed. The tissue response and biomechanics were evaluated at a single time point, whereas evaluation over time could provide insights into the potential effects of the scaffolds on different phases of wound healing. The outcomes of the study, especially the gross anatomical outcomes such as exposures, can be affected by external factors such as the learning curve of the surgeon, and animal species used in the study. Although the vaginal sheep model offers insight into host response and in vivo degradation, the forces applied to the vagina and pelvic floor in sheep differ from humans based on quadrupedalism. Despite these differences in posture, a sheep model is considered beneficial to evaluate human‐like host responses in pelvic floor research.^[^ ^13^, ^49^ ^]^ In current literature, many different electrospun materials are reported for use in pelvic floor repair, but results have scarcely led to use of large animal models, let alone humans. Evaluating our results to smaller animal‐ or even in vitro models has its limits in terms of comparability.

On the other hand, to our knowledge, this is the only preclinical evaluation of electrospun, degradable, E2‐releasing scaffolds in a sheep vaginal model. This step is necessary before clinically implementing new materials in POP treatment. Moreover, the used scaffolds are well characterized based on in vitro evidence.^[^ ^15^ ^]^ We added the internal vaginal control group from the same sheep, which allowed for retrospective comparison to short‐term sheep study of knit P4HB, and comparison between implant surgery and NTR. We studied not only biomechanical, but also clinical outcomes as erosion and infection on site. These records will be beneficial to aid sample size calculation for future in vivo or clinical safety studies. However, it should be noted that this study is not powered to evaluate complications.

## 5 Conclusion

Both ES P4HB and ES P4HB‐E2 scaffolds improved host response and provided the support to the vaginal wall tissue. This was evidenced by enhanced mechanical properties and increased macrophage infiltration after implantation compared to NTR and control tissue. Both scaffolds resulted in good tissue integration without signs of infection. Nonetheless, this study does not provide strong evidence for the beneficial effects of E2 incorporation, since no statistical differences were found between biomechanics and host response of ES P4HB and ES P4HB‐ES scaffolds. Even though, further optimization of ES P4HB‐E2 could enhance its benefits, it could be concluded that the incorporation of E2 may not be necessary, as ES‐P4HB scaffolds have already improved tissue integration and healing without E2, thanks to their ECM‐mimicking structure. In addition, electrospun P4HB scaffolds provided support to the vaginal tissue. ES P4HB scaffolds, as absorbable implants, can benefit patients by reinforcing the pelvic floor in POP repair. It is imperative to conduct further long‐term studies on ES P4HB to fully validate these effects and ensure their safety and efficacy in a clinical setting.

## Conflict of Interest

The authors declare no conflict of interest.

## Author Contributions

K.R.: writing the original manuscript, S.J.: performed the implantation and explantation surgeries, L.K.: performed the implantation and explantation surgeries, A.G.: performed explantation, data collection, and analyses, A.O.: scaffold preparation, J.P.R.: study design, writing, and editing, Z.G.: study design, performed explantation, data collection and analyses, methodology, writing and editing, grant acquisition. All authors revised the manuscript and approved the final version.

## Supporting information



Supporting Information

## Data Availability

The data that support the findings of this study are available from the corresponding author upon reasonable request.

## References

[1] L. G. O. Brito , G. M. V. Pereira , P. Moalli , O. Shynlova , J. Manonai , A. Y. Weintraub , J. Deprest , M. A. T. Bortolini , Int. Urogynecol. J. 2022, 33, 15. 34351465 10.1007/s00192-021-04953-1

[2] J. A. Deprest , R. Cartwright , H. P. Dietz , L. G. O. Brito , M. Koch , K. Allen‐Brady , J. Manonai , A. Y. Weintraub , J. W. F. Chua , R. Cuffolo , F. Sorrentino , L. Cattani , J. Decoene , A.‐S. Page , N. Weeg , G. M. Varella Pereira , M. G. M. C. Mori da Cunha de Carvalho , K. Mackova , L. H. Hympanova , P. Moalli , O. Shynlova , M. Alperin , M. A. T. Bortolini , Int. Urogynecol. J. 2022, 33, 1699.35267063

[3] J. M. Wu , A. Kawasaki , A. F. Hundley , A. A. Dieter , E. R. Myers , V. W. Sung , Am. J. Obstet. Gynecol. 2011, 205, 230. 10.1016/j.ajog.2011.03.046 PMC3630997 21600549

[4] F. J. Smith , C. D. Holman , R. E. Moorin , N. Tsokos , Obstet. Gynecol. 2010, 116, 1096. 20966694 10.1097/AOG.0b013e3181f73729

[5] Z. Guler , J. P. Roovers , Biomolecules 2022, 12, 94. 35053242 10.3390/biom12010094 PMC8773530

[6] FDA takes action to protect women's health, orders manufacturers of surgical mesh intended for transvaginal repair of pelvic organ prolapse to stop selling all devices, https://www.fda.gov/news‐events/press‐announcements/fda‐takes‐action‐protect‐womens‐health‐orders‐manufacturers‐surgical‐mesh‐intended‐transvaginal (accessed: March 2023).

[7] A. Dabica , O. Balint , F. Olaru , C. Secosan , L. Balulescu , S. Brasoveanu , M. Pirtea , D. Popin , I. F. Bacila , L. Pirtea , J. Pers. Med. 2024, 14, 622. 38929843 10.3390/jpm14060622 PMC11205245

[8] E. Yeung , K. Baessler , C. Christmann‐Schmid , N. Haya , Z. Chen , S. A. Wallace , A. Mowat , C. Maher , Cochrane Database Syst. Rev. 2024, 3, CD012079. 38477494 10.1002/14651858.CD012079.pub2 PMC10936147

[9] C. M. Glazener , S. Breeman , A. Elders , C. Hemming , K. G. Cooper , R. M. Freeman , A. R. Smith , F. Reid , S. Hagen , I. Montgomery , M. Kilonzo , D. Boyers , A. McDonald , G. McPherson , G. MacLennan , J. Norrie , Lancet 2017, 389, 381. 28010989 10.1016/S0140-6736(16)31596-3

[10] A. Mahdy , D. Karp , G. W. Davila , G. M. Ghoniem , Int. Braz. J. Urol. 2013, 39, 506. 24054379 10.1590/S1677-5538.IBJU.2013.04.08

[11] R. Ramanah , J. Mairot , M. C. Clement , B. Parratte , R. Maillet , D. Riethmuller , Int. Urogynecol. J. 2010, 21, 1151. 20424823 10.1007/s00192-010-1153-x

[12] SCENIHR (Scientific Committee on Emerging and Newly Identified Health Risks) , The safety of surgical meshes used in urogynecological surgery, European Commission, Luxembourg, 2015 https://ec.europa.eu/health/scientific_committees/emerging/docs/scenihr_o_049.pdf.

[13] C. M. Diedrich , Z. Guler , L. Hympanova , E. Vodegel , M. Zündel , E. Mazza , J. Deprest , J. P. Roovers , BJOG 2022, 129, 1039. 34865300 10.1111/1471-0528.17040 PMC9303173

[14] Z. Guler , L. A. Kaestner , E. Vodegel , L. Ras , S. Jeffrey , J. P. Roovers , Int. Urogynecol. J. 2024, 35, 713. 38430238 10.1007/s00192-023-05720-0 PMC11024044

[15] K. Verhorstert , A. Gudde , C. Weitsz , D. Bezuidenhout , J. P. Roovers , Z. Guler , ACS Appl. Bio. Mater. 2022, 5, 5270. 10.1021/acsabm.2c00691 PMC9682484 36315937

[16] S. G. Hansen , M. B. Taskin , M. L. Chen , L. Wogensen , J. V. Nygaard , S. M. Axelsen , J. Biomed. Mater. Res. B 2020, 108, 48. 10.1002/jbm.b.34364 30888115

[17] S. H. Laursen , S. G. Hansen , M. B. Taskin , M. Chen , L. Wogensen , J. V. Nygaard , S. M. Axelsen , J. Biomed. Mater. Res. B 2023, 111, 392. 10.1002/jbm.b.35158 PMC10087977 36075108

[18] M. G. M. C. Mori da Cunha , B. Arts , L. Hympanova , R. Rynkevic , K. Mackova , A. W. Bosman , P. Y. W. Dankers , J. Deprest , Acta Biomater. 2020, 106, 82. 32006652 10.1016/j.actbio.2020.01.041

[19] C. Glindtvad , M. Chen , J. Vinge Nygaard , L. Wogensen , A. Forman , C. C. Danielsen , M. B. Taskin , K.‐E. Andersson , S. M. Axelsen , J. Biomed. Mater. Res. B 2018, 106, 680. 10.1002/jbm.b.33875 28306194

[20] M. Krause , T. L. Wheeler 2nd , T. E. Snyder , H. E. Richter , J. Pelvic Med. Surg. 2009, 15, 105. 22229022 10.1097/SPV.0b013e3181ab4804 PMC3252029

[21] C. M. Diedrich , A. W. Kastelein , F. M. Verri , M. A. Weber , C. Ince , J. W. R. Roovers , Neurourol. Urodyn. 2019, 38, 1298. 30947367 10.1002/nau.23977 PMC6850718

[22] N. Mangir , C. J. Hillary , C. R. Chapple , S. MacNeil , Eur. Urol. Focus 2019, 5, 280. 28753895 10.1016/j.euf.2017.05.004

[23] E. V. Vodegel , A. W. Kastelein , C. H. J. R. Jansen , J. Limpens , S. E. Zwolsman , J‐P. W. R. Roovers , C. R. Hooijmans , Z. Guler , Neurourol. Urodyn. 2022, 41, 115. 34643282 10.1002/nau.24819 PMC9293291

[24] C. M. Diedrich , J. P. Roovers , T. H. Smit , Z. Guler , Mater. Sci. Eng. C 2021, 120, 111702. 10.1016/j.msec.2020.111702 33545861

[25] J. Cremer , B. P. Kaltschmidt , A. Kiel , J. Eberhard , S. Schmidt , C. Kaltschmidt , B. Kaltschmidt , A. Hütten , D. Anselmetti , Polymers 2023, 15, 1247. 36904487 10.3390/polym15051247 PMC10006934

[26] S. Todros , P. G. Pavan , A. N. Natali , J. Biomed. Mater. Res. B 2017, 105, 689. 10.1002/jbm.b.33586 26671827

[27] I. Keridou , L. Franco , L. J. Del Valle , J. C. Martinez , L. Funk , P. Turon , J. Puiggali , Polymers 2020, 12, 2024. 32899844 10.3390/polym12092024 PMC7564121

[28] C. R. Deeken , D. C. Chen , M. Lopez‐Cano , D. P. Martin , A. Badhwar , Front. Surg. 2023, 10, 1157661. 37123542 10.3389/fsurg.2023.1157661 PMC10130450

[29] L. Hympánová , R. Rynkevic , S. Román , M. G. M. C. Mori da Cunha , E. Mazza , M. Zündel , I. Urbánková , M. R. Gallego , J. Vange , G. Callewaert , C. Chapple , S. MacNeil , J. Deprest , Eur. Urol. Focus 2020, 6, 190. 30049658 10.1016/j.euf.2018.07.024

[30] M. Mori da Cunha , L. Hympanova , R. Rynkevic , T. Mes , A. W. Bosman , J. Deprest , Materials 2019, 12, 1174. 30974868 10.3390/ma12071174 PMC6480159

[31] H. Hjort , T. Mathisen , A. Alves , G. Clermont , J. P. Boutrand , Hernia 2012, 16, 191. 21972049 10.1007/s10029-011-0885-y PMC3895198

[32] S. Manodoro , M. Endo , P. Uvin , M. Albersen , J. Vlcil , A. Engels , B. Schmidt , D. De Ridder , A. Feola , J. Deprest , BJOG 2013, 120, 244. 23240803 10.1111/1471-0528.12081

[33] K. W. J. Verhorstert , A. N. Gudde , B. S. Kortz , J. Limpens , J. W. R. Roovers , C. R. Hooijmans , Z. Guler , Neurourol. Urodyn. 2021, 40, 1107. 33951222 10.1002/nau.24677 PMC8359983

[34] L. Hympanova , M. G. M. C. Mori da Cunha , R. Rynkevic , M. Zündel , M. R. Gallego , J. Vange , G. Callewaert , I. Urbankova , F. Van der Aa , E. Mazza , J. Deprest , J. Mech. Behav. Biomed. Mater. 2017, 74, 349. 28668592 10.1016/j.jmbbm.2017.06.032

[35] K. K. Shapiro , K. M. Knight , R. Liang , J. Cook , G. E. King , S. D. Abramowitch , P. A. Moalli , Am. J. Obstet. Gynecol. 2021, 224, 78. 10.1016/j.ajog.2020.07.005 32707267

[36] K. M. Knight , G. E. King , S. L. Palcsey , A. Suda , R. Liang , P. A. Moalli , Acta Biomater. 2022, 148, 323. 35671876 10.1016/j.actbio.2022.05.051 PMC9453339

[37] T. J. Sill , H. A. von Recum , Biomaterials 2008, 29, 1989. 18281090 10.1016/j.biomaterials.2008.01.011

[38] I. Jun , H. S. Han , J. R. Edwards , H. Jeon , Int. J. Mol. Sci. 2018, 19, 745. 29509688 10.3390/ijms19030745 PMC5877606

[39] M. Vashaghian , B. Zandieh‐Doulabi , J. P. Roovers , T. H. Smit , Tissue Eng., Part A 2016, 22, 1305. 27676643 10.1089/ten.TEA.2016.0194

[40] S. Mukherjee , S. Darzi , A. Rosamilia , V. Kadam , Y. Truong , J. A. Werkmeister , Biomacromolecules 2019, 20, 454. 30512928 10.1021/acs.biomac.8b01661

[41] D. O'Shaughnessy , D. Grande , D. El‐Neemany , S. Sajjan , N. Pillalamarri , D. Shalom , H. Winkler , Int. Urogynecol. J. 2022, 33, 2213. 34125243 10.1007/s00192-021-04851-6

[42] Z. Strizova , I. Benesova , R. Bartolini , R. Novysedlak , E. Cecrdlova , L. K. Foley , I. Striz , Clin. Sci. 2023, 137, 1067. 10.1042/CS20220531 PMC10407193 37530555

[43] P. J. Murray , J. E. Allen , S. K. Biswas , E. A. Fisher , D. W. Gilroy , S. Goerdt , S. Gordon , J. A. Hamilton , L. B. Ivashkiv , T. Lawrence , M. Locati , A. Mantovani , F. O. Martinez , J.‐L. Mege , D. M. Mosser , G. Natoli , J. P. Saeij , J. L. Schultze , K. A. Shirey , A. Sica , J. Suttles , I. Udalova , J. A. Ginderachter van , S. N. Vogel , T. A. Wynn , Immunity 2014, 41, 14. 25035950 10.1016/j.immuni.2014.06.008 PMC4123412

[44] S. Shafaat , S. Roman Regueros , C. Chapple , S. MacNeil , V. Hearnden , J. Tissue Eng. 2023, 14, 20417314221149207. 36726532 10.1177/20417314221149207 PMC9885031

[45] F. Klingberg , B. Hinz , E. S. White , J. Pathol. 2013, 229, 298. 22996908 10.1002/path.4104 PMC4005341

[46] Y. P. Chen , T. S. Lo , Y. H. Chien , Y. H. Kuo , S. J. Liu , Polymers 2024, 16, 1667. 38932015 10.3390/polym16121667 PMC11207985

[47] S. Shafaat , N. Mangir , S. R. Regureos , C. R. Chapple , S. MacNeil , Neurourol. Urodyn. 2018, 37, 716. 29439287 10.1002/nau.23510

[48] Y. M. Liu , K. W. Choy , W. T. Lui , M. W. Pang , Y. F. Wong , S. K. Yip , Hum. Reprod. 2006, 21, 303. 16155073 10.1093/humrep/dei296

[49] E. V. Vodegel , Z. Guler , L. Ras , K. Mackova , A. C. H. M. Groeneveld , D. Bezuidenhout , J. Deprest , S. T. Jeffery , J.‐P. W. R. Roovers , Int. J. Gynaecol. Obstet. 2023, 162, 1042. 37151087 10.1002/ijgo.14816

