# Supplementary material for: Evaluation of Electrospun Poly‐4‐Hydroxybutyrate as Biofunctional and Degradable Scaffold for Pelvic Organ Prolapse in a Vaginal Sheep Model
Source: Macromol Biosci. 2025 Feb 26;25(4):2400412. doi: 10.1002/mabi.202400412 (PMC11995834; doi:10.1002/mabi.202400412)
Supplement: Supplementary file 1 — Supporting Information [file MABI-25-2400412-s001.docx]

EVALUATION of ELECTROSPUN POLY-4-HYDROXYBUTYRATE as BIOFUNCTIONAL and DEGRADABLE SCAFFOLD for PELVIC ORGAN PROLAPSE in a VAGINAL SHEEP MODEL

Krista L.C. van Rest, Stephen T. Jeffrey, Lisa Kaestner, Aksel Gudde, Anel Oosthuysen, Jan-Paul W.R. Roovers, Zeliha Guler.

**Supplementary material**

1. Porosity and hydrophilicity measures of the ES P4HB and ES P4HB-E2 scaffolds

The porosity of the scaffolds was measured using the differential mass of the scaffold in air versus the scaffold mass in a heptane solution and calculated using the following equation:

ϕ=1-((m_air-m_heptane))/(p_heptane*V_total )

Where m_air and m_heptane are the masses of the samples in air and suspended in heptane respectively, ρ_heptane is the density of heptane, and V_total is the bulk density of the porous sample.

Water contact angle values of the scaffolds were measured to by placing water droplets (5 microlitres) onto the surface of the P4HB scaffolds (n=5). Images were taken of the droplets and the static water contact angles were measured using open source image analyses software (Fiji)(1).

1. Sample size calculation

Sample size calculated based on two-sided Fisher exact test with 5% significance level, 80% power to detect of difference between 10-90% with as quantitative endpoint the explant stiffness of comfort zone (N/mm) and control. Stiffness at comfort zone is a quantifiable vaginal mechanical property, which is important in development of pelvic organ prolapse and implantation reconstructive surgery. To detect difference (50% difference is expected between the vaginal tissue after implant surgery and native tissue repair, based on the results obtained from six-month data (2)) 6 animals per treatment group were required. For control group 4 sheep was used as the differences between comfort zone stiffness of the vaginal tissue after NTR is not expected to be higher than 30%, based on the previous studies). To reduce the animal number, the “unexpected loss of 10%” per group was not take into account as the study duration is only 3-months and we didn’t encounter any adverse effects in the previous sheep studies we conducted(2-4). Therefore 6 animal per biomaterial group (2 groups) and 4 animals for control group (NTR), per time point (1 time points) is required (6 x 2 + 4) = 16 animals in total).

1. Surgical procedures

All sheep had vaginal wall reconstruction of the posterior compartment, six with ES P4HB, six with ES P4HB-E2 and four underwent NTR. To further reduce the animal number, middle vaginal tissue (vaginal control) was collected of the same sheep as controls. The surgeon was blinded for treatment group at start of surgery. NTR was started with 30 mm midline vaginal epithelium incision and blunt dissection of the recto-vaginal connective tissue. This fascial structure was plicated with three interrupted 3/0 polyglactin 910 (Vicryl, Ethicon, Raritan, USA) sutures. The vaginal wall was closed with a running 3/0 polyglactin 910 suture.

During NTR of the vaginal posterior compartment, the surgeon was informed by a researcher to continue NTR or place an implant (without knowing its content). Our earlier report (Diedrich et al, supplementary material 1.(2)) has detailed descriptions of the surgical procedure with the same method. In short, all animals were pre-medicated with midazolam 0.3mg/kg and ketamine 3mg/kg, and received antibiotic prophylaxis, before general anesthesia with intubation was admitted, in dorsal decubitus position. Before start of the surgical procedure, bladder and rectum were emptied, surgical site was sheared, disinfected and covered with surgical sterile drapes.

Before incision, the surgical area was prepared with retractor and hooks for better access. To start the procedure, the posterior vaginal wall approximately 3 cm cranially from the hymen was lifted. The rectovaginal septum was dissected by aqua-dissection, and incisions were made above the hymeneal ring. (Implant) space was bluntly and sharply created between the vaginal epithelium and rectal serosa. If indicated, the dry mesh implant was measured before implantation, then positioned and fixed with polypropylene sutures. All vaginal walls were closed with continuous polyglactin 910 suture. Thereafter, a vaginal tampon was inserted for 24 hours. Postoperative analgesia was administered with meloxicam, buprenorphine and chlorocresol up to 3 days after the procedure. Observation of the animals was carried out for 1 week post-surgery and surgical site was inspected regularly. At 3 months post-surgery, sheep underwent gross necropsy and thereafter were euthanized.

1. In vivo degradation profile of P4HB explants

Vaginal tissue digestion: P4HB explants were incubated overnight with collagenase (type I, from Clostridium histolyticum) solution (1.0mg/mL) in TESCA buffer (50mM TES, 2mM CaCl2, 10mM NaN3, pH 7.4) at 37°C shaker (600 rpm/min) and were then rinsed with sterile water, 70% ethanol and air dried.

Gel permeation chromatography (GPC): P4HB scaffold samples were dissolved at 1mg/mL in chloroform, filtered using a 0.45 𝜇m filter to remove undigested particulates, and 95 𝜇L of this solution was then injected onto a GPC column. GPC was performed in chloroform at 1ml/min using a Polymer Labs, PLgel column (5micron, mixed C, 300 × 7.5 mm) with an Agilent 1100 Series HPLC with RI detector. Calibration was conducted against monodisperse polystyrene standards. Molecular weight is reported in units of Daltons (Da).

Scanning electron microscopy (SEM): After tissue removal, P4HB scaffolds were coated with a layer of gold about 3 nm thick in an EMITECH K650X sputter coater and examined at room temperature in a JEOL JSM6700F field emission scanning electron microscope at an accelerating voltage of 2.5 kV.

1. Histomorphology

For histomorphology analyses, the same method for sample preparation and scoring was used to our short-term in vivo study, described by Diedrich et.al.(2). The piece of explant for histology and IHC was fixed in 10% neutral buffered formalin overnight, washed in phosphate-buffered saline for 2 hours and stored in Ethanol 70%. After embedment in paraffin, pieces were cut into 5 µm longitudinal slices to create a specimen that included implant (if present), interface and surrounding native tissue. Various staining was performed on all slices. Specimens were tested for presence of foreign body giant cells (FBGC), polymorphonuclear cells (PMN), vessels, collagen and extracellular connective tissue through haematoxylin and eosin (H&E) and Masson’s trichrome staining. Verhoeff-Van Gieson’s stain was used for presence of elastin. With immunohistochemistry staining, the presence of smooth muscle content (αSMA), neovascularization (CD34), macrophages type I (HLA-DR) and macrophages type II (CD-163) were tested.

Every explant was examined by independent researchers and one pathologist was consulted in case there is a disagreement in the scoring; one researcher prepared the electronic histology slides by selecting 5 different, representative locations on each slice of the specimen at a magnification of x400, and ensuring a code for each slide. Locations were selected at the interface of the implant and its surrounding tissue. The other two blinded researchers examined and scored all coded slides individually without knowing the allocated group of the specimen.

For H&E stain, the score indicates the number of counted cells/vessels per high power field by an ordinal scale (0 = none, 1 = 1-5 cells/vessels, 2 = 6-10 cells/vessels, 3 = >10 cells/vessels). For Masson’s trichome stain, that identifies extracellular connective tissue (mainly unspecified collagen), and for Verhoeff-Von Gieson’s stain, that identifies elastin, a semi-quantitative method was used to score all slides. The scores for these slide are; 0 = absence, 1 = mild presence, 2 = large presence, 3 = abundance, 4 = great abundance. For each explant a mean score per presence was calculated by adding all scores of 5 slides and dividing these by 5.

M2/M1 ratio was calculated by dividing scores for presence of M2 and M1. On alpha-SMA images, smooth muscle and vessels were excluded from evaluation.

For IHC staining, sections were processed with a mouse and rabbit specific HRP/DAB (ABC) Detection IHC Kit (Abcam) and three different monoclonal antibodies (**Table S1**). Antibodies were diluted in 1% bovine albumin serum (BSA) (Sigma-Aldrich). Semi-quantitative assessment of the extent of immunostaining was performed using a qualitative grading scale; absent=0, mild presence=1, large presence=2, abundance=3, great abundance=4 (**Figure S1)**. **Figure S2** shows negative controls for IHC staining to compare with **Figure 6.** in the main manuscript.

**Table S1***.* (Cambridge, UK), DAKO (Glostrup, Denmark), AbD Serotec (Kidlington, UK)

| Antibody | Concentration | Company |
| --- | --- | --- |
| mouse anti-smooth muscle actin (SMA) | 1:200 | DAKO |
| mouse anti-HLA-DR | 1:100 | Abcam |
| mouse anti-CD163 | 1:200 | AbD Serotec |

**Figure S1**. Representative images of slides with immunostaining for semi-quantitative assessment, presenting macrophages (A-D = scores 1-4) and alpha-SMA (E-H = scores 0-3). Images F and G show unorganized alpha-SMA, and H shows organized alpha-SMA.


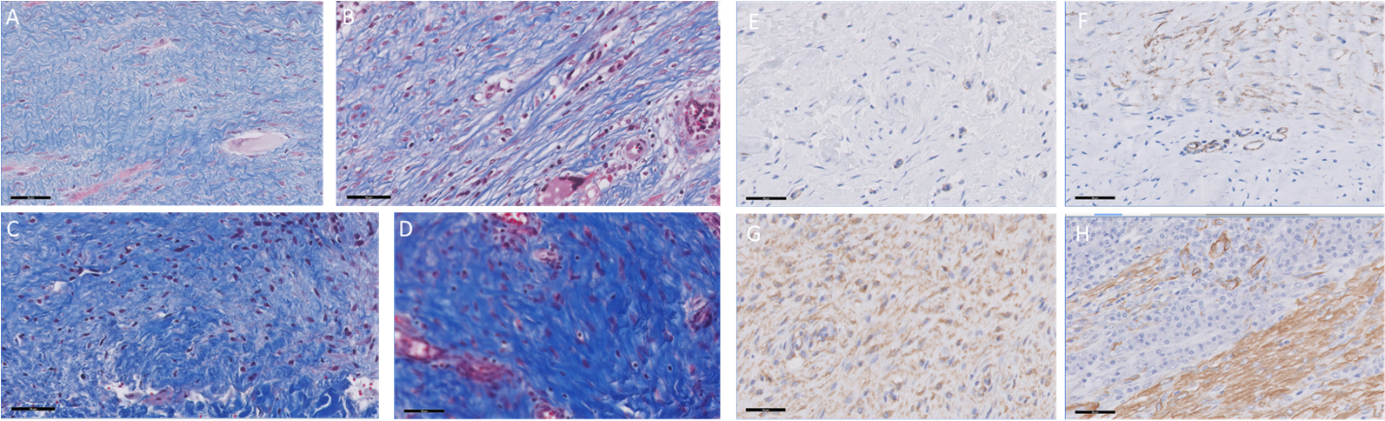


**Figure S2.** Representative images of negatives of slides with immunostaining for CD 163 (A) and HLA DR (B).


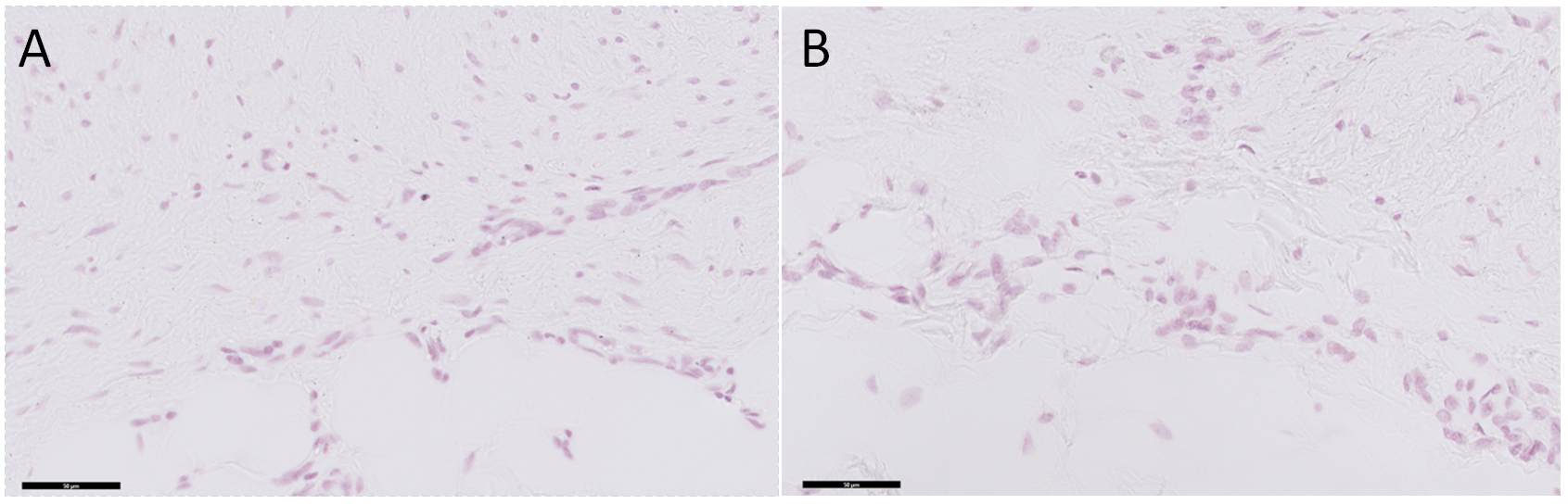


1. Tissue dehydration for scanning electron microscopy

The tissue samples (explants) were rinsed in water for 10 mins. Then the tissue samples were dehydrated in gradual alcohol; 70% ethanol (15 mins), 80% ethanol (20 mins), 90% ethanol (20 mins), 96% ethanol (20 mins) by repeating each step three times. Finally, the samples were soaked in 100% ethanol (30 mins) twice and incubated in HMDS (for 30 mins) and air-dried.

**References**

1. Schindelin J, Arganda-Carreras I, Frise E, Kaynig V, Longair M, Pietzsch T, et al. Fiji: an open-source platform for biological-image analysis. Nat Methods. 2012;9(7):676-82.

2. Diedrich CM, Guler Z, Hympanova L, Vodegel E, Zundel M, Mazza E, et al. Evaluation of the short-term host response and biomechanics of an absorbable poly-4-hydroxybutyrate scaffold in a sheep model following vaginal implantation. BJOG. 2022;129(7):1039-49.

3. Guler Z, Kaestner LA, Vodegel E, Ras L, Jeffrey S, Roovers JP. Two-Year Preclinical Evaluation of Long-Term Absorbable Poly-4-hydroxybutyrate Scaffold for Surgical Correction of Pelvic Organ Prolapse. Int Urogynecol J. 2024;35(3):713-22.

4. Vodegel EV, Guler Z, Ras L, Mackova K, Groeneveld A, Bezuidenhout D, et al. Vaginal changes after ovariectomy in ewes: A large animal model for genitourinary syndrome of menopause. Int J Gynaecol Obstet. 2023;162(3):1042-9.
